# Course of macroscopic anatomy in Magdeburg under pandemic conditions

**DOI:** 10.3205/zma001358

**Published:** 2020-12-03

**Authors:** Erik Wolniczak, Thomas Roskoden, Hermann-Josef Rothkötter, Silke D. Storsberg

**Affiliations:** 1 Otto-von-Guericke-Universität, Medizinische Fakultät, Institut für Anatomie, Magdeburg, Germany

**Keywords:** anatomy, teaching methods, e-learning, pandemic, CoVid-19

## Abstract

**Introduction and objectives: **The Covid-19 pandemic has created major challenges for university teaching. At the beginning of the summer semester 2020, teaching at the Medical Faculty in Magdeburg was almost completely online. Also the course in macroscopic anatomy had to be replaced by virtual e-learning offers.

**Methods: **Videos and photo presentations of the preparation steps and structures to be displayed were made available online. The reactions of the students showed very quickly that the three-dimensionality, the independent preparation and the haptics of the object to be studied make up a large part of this subject.

**Results and conclusions:** Virtual e-learning offerings are a useful supplement to, but not a substitute for, active dissecting on body donors. By changing the course offerings in compliance with hygiene and distance rules, we were able to offer a classroom course again during the semester, which was expressly welcomed by the students.

## Zusammenfassung

**Einleitung und Zielsetzung: **Die Covid-19-Pandemie hat die Lehre in der Universität vor große Herausforderungen gestellt. Der Lehrbetrieb der Medizinischen Fakultät in Magdeburg wurde zu Beginn des Sommersemesters 2020 fast vollständig online durchgeführt. Auch der Kursus der makroskopischen Anatomie musste zunächst durch virtuelle E-Learning Angebote ersetzt werden.

**Methodik: **Videos und Fotopräsentationen der Präparierschritte und darzustellenden Strukturen wurden online zur Verfügung gestellt. Die Reaktionen der Studierenden zeigten sehr schnell, dass die Dreidimensionalität, das selbstständige Präparieren und die Haptik des zu studierenden Objektes dieses Fach zum Großteil ausmachen.

**Ergebnisse und Schlussfolgerungen: **Virtuelle E-Learning Angebote sind eine sinnvolle Ergänzung, aber kein Ersatz für das aktive Präparieren an Körperspendern. Durch Umstellung des Kursangebots unter Einhaltung von Hygiene- und Abstandregeln konnten wir im Laufe des Semesters wieder einen Präsenzkurs anbieten, was von den Studierenden ausdrücklich begrüßt wurde.

## Introduction

In the summer semester 2020, about 190 students were enrolled in the first year of study at the Otto-von-Guericke-University Magdeburg. In the macroscopic anatomy course, 1 seminar group (approx. 20 students) prepares on 2 consecutive days on 2 body donors in the "parallel group system". While in the winter semester 2019/20 the students did the preparation themselves, in the first 4 weeks of the summer semester the anatomical teaching contents were made available alternatively with digital methods. The lectures were held as video conferences (ZOOM) with additional lecture notes [https://moodle.med.ovgu.de/login/login.php]. 

## Project description

For the practical part, the structures to be depicted were prepared by the lecturers, supported by the preparators. The preparation state was documented with various camera systems (smartphone, digital camera, action camera, etc.). The action camera was mounted on a helmet in order to provide the viewer with a first-person perspective. 

Videos and photo presentations were made available on the university‘s internal online platform (Moodle) with restricted access. The institute's own preparation instructions (*Anleitung zum Präparierkurs und Hirnkurs an der Medizinischen Fakultät der Otto-von-Guericke-Universität Magdeburg*, manuscript, 18^th^ ed., 2019 [1]) served as a temporal orientation of the procedure. The photo presentations contained captions and comments, the videos received audio comments and the structures were displayed with text. In total, the students had 49 videos and 11 presentations at their disposal [https://moodle.med.ovgu.de/login/login.php].

In addition to scheduling, viewing and editing the videos and photos for the presentations, a digital infrastructure was also set up. 

## Methods

There were 2 meetings of the involved parties scheduled weekly. At the beginning of the week the schedule was planned. At the weekend the results were reviewed and made available online [https://moodle.med.ovgu.de/login/login.php]. 

The advantages and disadvantages of the procedure are described below:

### Pro (lecturer)

- more detailed preparation and representation of the structures
- use of different, alternative preparation methods (different from the preparation instructions)
- extended further training opportunities for lecturers
- result videos and photos can be used permanently as digital support for future vintages

#### Contra (lecturer)

- approx. 3 times the time required! (interindividual differences)
- hardly any personal interaction with students
- videos only refer to preparations of this semester, transferability to “new” preparations sometimes difficult
- despite limited access to the online platform (Moodle), digital distribution cannot be completely controlled, therefore clear marking of the material with faculty logo and institute identification

#### Pro (student)

- focal points for students clearly defined through videos/presentations
- result videos and photos can be used asynchronously, fast forward/rewind possible
- traceability of individual preparation steps (often not possible due to “parallel groups” in presence mode)

#### Contra (student)

- spatiality and haptics of the real specimen are missing
- the possibility to represent structures yourself is missing (“sense of achievement”)
- joint learning with fellow students is missing
- anatomical variability of body donors can only be mediated to a limited extent

Since 06.05.2020, restricted classroom teaching has been possible again, provided that the applicable hygiene measures are observed [https://ms.sachsen-anhalt.de/themen/gesundheit/aktuell/coronavirus/]. Lectures continued to take place online. A maximum of 50 students (instead of 100) were present during the dissection course. The starting times of the course were staggered for 2 subgroups (25 persons each) to allow a certain distance between the students in the changing rooms. The Institute provided protective clothing (surgical gowns) and mouth and nose protectors for single use; the number of students per body donor was limited to 5. The limitation of the number of participants halved the course time for the students, but short seminars were increasingly offered in each course and extended self-study periods with lecturers. This meant that there was no need to change tables or mix the groups and the students were able to deal with other preparations to a much lesser extent. Lists of participants would have been available for tracking possible infection chains.

## Results

Before the last audit of the semester, a survey was conducted among students to find out how helpful the materials we provided were perceived. The survey showed that 95% of the 148 students who participated in the survey found the materials on Moodle to be “very helpful” or “helpful” and only 2 students had not taken advantage of this offer. 

Overall, the following conclusions can be drawn:

### Positive

- high appreciation of active dissection (“...a dissection course cannot be replaced...”)
- own preparation clearly promotes learning effect (“...is eminent to be able to imagine the structures and their topology in three dimensions...”)
- more intensive support through smaller groups
- better preparation of audit certificates through intensified seminars
- Risk of infection minimized, but not eliminated!

#### Negative

- little knowledge about other preparations and varieties
- overall shortened individual preparation time with greater effort on the part of the lecturers
- overall fear/care/uncertainty of the students
- expectations of students are very high, own preparation initiative decreases, the lecturer becomes a “preparation service provider”

## Conclusion

The majority of students clearly state that a virtual dissection course cannot replace the real dissection course. The entire college was able to gather a great deal of experience in order to be able to react adequately and provide the students with the best possible training under pandemic conditions. Based on these experiences (teaching and logistics), the teaching can be designed for the coming winter semester; in classroom, hybrid or online courses.

## Competing interests

The authors declare that they have no competing interests. 
